# Gynomorphic Mandible Morphology in the Dobsonfly, *Corydalus cornutus*

**DOI:** 10.1673/031.007.2301

**Published:** 2007-04-13

**Authors:** David E. Bowles, Atilano Contreras-Ramos, Robert W. Sites

**Affiliations:** ^1^ National Park Service, Heartland Inventory and Monitoring Program, c/o Department of Biology, Missouri State University, 901 South National Avenue, Springfield, MO 65897, USA.; ^2^ Centro de Investigaciones Biológicas, Universidad Autónoma del Estado Hidalgo, Apdo. Postal 1-69, Plaza Juárez, Pachuca, Hidalgo 42001, Mexico; ^3^ Enns Entomology Museum, Division of Plant Sciences, University of Missouri, Columbia, MO 65211, USA.

**Keywords:** *Corydalus cornutus*, mandible morphology, gynomorphism

## Abstract

Two aberrant males of *Corydalus cornutus* (L.) (Insecta: Megaloptera), which exhibit unusually short mandibles with discrete dentition, are recorded from a locality in Missouri. Morphological details of the specimens, as well as implications for the overall morphological variation of the genus and species are discussed. The term gynomorphic is suggested as the best descriptor of this case, given that little explanatory information is available to classify these specimens as true gynandromorphs.

## Introduction

**Figures 1–4.**
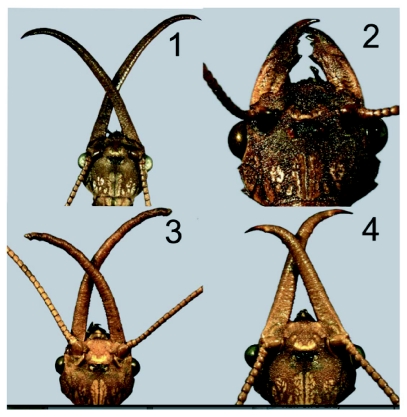
Male and female *Corydalus cornutus* showing mandible morphology. 1. Typical male, 2. Typical female, 3. Male with long mandibles, Jefferson County, Missouri, 4. Male with relatively short mandibles, Cooper County, Missouri.

*Corydalus cornutus* (L.) is the most common and widely distributed of three species in this genus of dobsonflies known to occur in the United States, and it is characterized by the males having elongate mandibles without teeth (Contreras-Ramos 1998). Males of several species of *Corydalus* have mandibles that are considerably long relative to head width whereas those of other species are short and female-like in appearance. Female mandibles are unmodified except in one South American species (Contreras-Ramos 1998). The length of female mandibles typically is subequal to head width with four distinct teeth (3 preapical, 1 apical) (Contreras-Ramos 1998). In his phylogenetic analyses of the Megaloptera, Contreras-Ramos (1998, 2004) showed that short, female-like mandibles among males of *Corydalus* species represent the plesiomorphic character state whereas the elongated mandibles of other species are apomorphic. Males of several South American species (*e.g., C. arpi* Navás, *C. cephalotes* Rambur, *C. hecate* MacLachlan, and *C. ignotus* Contreras-Ramos) exhibit unmodified, female-like mandibles (Contreras-Ramos 1998). Other species exhibit a transitional state of elongate mandibles with a discrete and well-developed dentition (*e.g., C. colombianus* Contreras-Ramos). Among species with elongate mandibles, they tend to be proportionately short (*e.g., C. texanus* Banks), or long (*e.g., C. cornutus* (L.)). However, some species have a broad range of variation (*e.g., C. nubilus* Erichson). In the case of *C. cornutus*, despite long mandibles being the most common condition, there may be apparent “transitional states” that are intermediate between those of females and males with elongate mandibles (Contreras-Ramos 1998).

**Figures 5–8.**
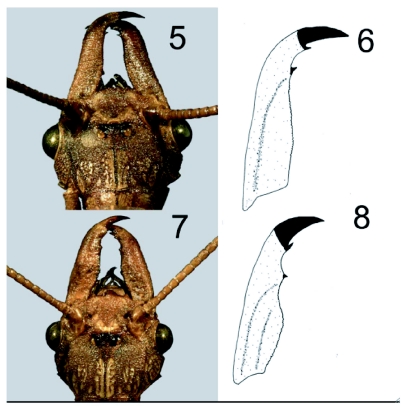
Examples of *Corydalus cornutus* exhibiting aberrant mandible morphology. 5. Male, head, Jefferson County, Missouri. 6. Line drawing of left mandible. 7. Second male, head, Jefferson County, Missouri. 8. Line drawing of left mandible.

A series of *Corydalus cornutus* was discovered in the Enns Entomology Museum, University of Missouri-Columbia (UMC) in which two of three males had unusually short mandibles with sufficient dentition so as to give them a superficially female appearance. Herein, the mandibles of these unusual specimens are illustrated, and possible explanations for these morphological variants are discussed.

## Materials and Methods

Terminalia of pinned specimens were removed and soaked in lactophenol for 24 hours, cleared in warm 10% potassium hydroxide, and rinsed in 70% isopropyl alcohol to remove any remaining residue. Cleared terminalia were stored in glycerin-filled genitalia vials attached to the specimen pins. Identification of adult specimens is based on Contreras-Ramos (1998). Head capsule widths and mandible lengths were measured (mm) at 10X magnification with a calibrated ocular grid. Forewing lengths were measured (mm) using a hand scale without magnification. Composite, continuous depth-of-field photographs were produced using Synchroscopy Automontage® software and Leica® microscopy. Specimens examined in this study are deposited in the UMC and in the collection of the senior author. Collection data inferred from incomplete label data (*i.e.*, where state and other identifying collection information was provided) are placed within brackets.

**Table 1.**
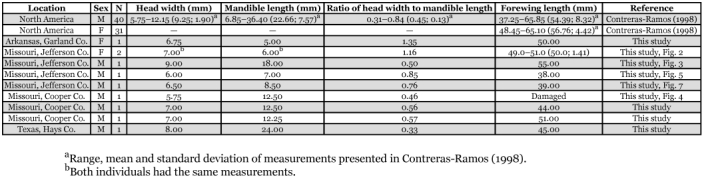
Comparison of measurements of head width, mandible length and forewing length for selected specimens of *Corydalus cornutus*.

## Material Examined

*Corydalus cornutus* (L.): Collected in Arkansas, Garland Co., Camp Clearfork, 15 mi W. Hot springs, XI.19.1994, D. E. Bowles, at light, female. Collected in Missouri, Jefferson Co., Barnhart, VI.1.37, 1 male; same data except VII.31.37, E. P. Meiners, 1 male; same data except VII.10.37, 1 male; same data except VII.7.37, 2 females; same data except [Cooper Co.], Boonville, Bell Orchard, VII.19.1951, W. R. Enns, 1 male; same data exept VII.21.1957, F. Wood, 1 male; same data except VII.19.1958, Enns, Wood, St Aubin, 1 male. Collected in Texas, Hays Co., San Marcos River, IX.28.[19]79, Colr. M. W., 1 male.

## Results and Discussion

Wings and genitalia of the specimens reported herein are consistent with those of typical *C. cornutus*, (Contreras-Ramos 1998). Typical males (Figure 1) and females (Figure 2) exhibited mean head width to mandible length ratios of 0.45 and >1.0, respectively (Table 1; Contreras-Ramos 1998). One male from Jefferson County, Missouri, had elongate, tubular mandibles without dentition (head width/mandible length=0.5) (Figure 3). However, two other males from that location had aberrant mandibles with size and dentition that suggests a female-like appearance (Figures 5–8). Specifically, these two males had head width to mandible length ratios of 0.76 and 0.85, which is midway (mean = 0.83) between those of the two females and the other male collected from the same location (Table 1). Perhaps more striking than the length of the mandibles of the aberrant specimens is that they are relatively broad, dorsoventrally flattened, and had clear dentition (1 apical and 2 preapical). The height of the teeth in these specimens were approximately half those of typical females. In addition, three males from Cooper County, Missouri also had relatively short mandibles (Figure 4, Table 1), but these specimens lack dentition, the mandibles are tubular in shape, and the ratio of head width to mandible length ranged from 0.46 to 0.57, which is comparable to typical males with elongated mandibles.

The condition of some *C. cornutus* males in which mandibles appear as transitional between those of females and males with highly elongate, dentition-free mandibles was described and illustrated by Contreras-Ramos (1998, Figs. 12I–12K). Although some of the male *C. cornutus* described by Contreras-Ramos had short mandibles with a tendency for the occurrence of teeth, none of these specimens was as extreme in female-like appearance as the two aberrant males from Missouri.

Gynandromorphs are sexually abnormal individuals with some parts genotypically and phenotypically male and other parts female, including secondary sexual characteristics (Lincoln et al. 1998). Gynandromorphism has been commonly reported from various groups within the Class Insecta. In contrast to gynandromorphism, the term gynomorphism has been used to describe males that have a morphological resemblance to females but not necessarily having female sexual characters (Lincoln et al. 1998). Gynomorphism has been less frequently reported in the literature and primarily used in reference to sex-related –coloration among the Odonata (Sirot and Brockmannn 2001, Sirot et al. 2003).

The aberrant males described here possibly could be viewed as gynandromorphs, but, because there is no evidence beyond the aberrant mandible morphology to indicate that these specimens fit this description, we suggest that they may simply be gynomorphic. Among males of other species of *Corydalus*, transitional mandible forms range widely from those that are female-like to those that are highly modified and elongate. Moreover, some species appear to be consistent in mandible type (either elongate with reduced dentition or short with discrete dentition), or particularly variable (*e.g., C. nubilus* Erichson). Occasionally, small males with short mandibles co-occur within populations with typical large “normal” males with long mandibles, as is documented herein. As males fight for females prior to copulation, implications regarding possible advantages or disadvantages for smaller males remain uncertain. Size variation in the body and mandibles of *Corydalus* species is an interesting phenomenon in which genetics, the environment, phylogeny, and other potential, but unknown, factors may play a role. Anthropogenic and environmental factors cannot be ruled out as affecting the observed aberrant mandible morphology. Both of the aberrant specimens described here were collected from one location in 1937, when environmental regulations in the United States were few and relatively weak. The aquatic larvae of the adults described here possibly could have been subjected to any number of physical and chemical disturbances that might have altered their normal development.

We have collectively examined several hundred specimens of *C. cornutus* from throughout the United States, but we have not previously observed specimens as aberrant as those described here. Clearly, such variation is rare, but additional descriptions of male *C. cornutus* with short mandibles may provide insight into the relative frequency of such occurrences as well as their significance.
